# Study of Preparation and Properties of Stereoregular Poly(cyclohexenylene carbonate)

**DOI:** 10.3390/molecules28135235

**Published:** 2023-07-05

**Authors:** Ming Zhang, Chengqian Zhang, Pengyuan Zhang, Zhengyong Liang

**Affiliations:** 1 State Key Laboratory of Coking Coal Resources Development and Comprehensive Utilization, Pingdingshan 467002, China; nyhgyjyzm@163.com; 2 School of Chemical Engineering, Zhengzhou University, Zhengzhou 450001, China; 1901329029@nyist.edu.cn (C.Z.); zhangpengyuan2526@163.com (P.Z.)

**Keywords:** carbon dioxide, cyclohexene oxide, poly(cyclohexenylene carbonate), asymmetric copolymerization, dinuclear cobalt complex

## Abstract

Fixing carbon dioxide as a polymer material is an effective and environmentally beneficial approach for reducing the harm of CO_2_ greenhouse gas. In this paper, carbon dioxide and cyclohexene oxide were used as co-monomers, and a chiral binuclear cobalt complex with a biphenyl linker was employed as the catalyst to successfully prepare a poly(cyclohexenylene carbonate) with high stereoregularity. The influence of catalyst structure, CO_2_ pressure, and operating temperature on the copolymerization rate and polymer structure were systematically investigated. Optimal catalyst structure and operating conditions were determined, resulting in an excellent poly(cyclohexenylene carbonate) with a stereoregularity as high as 93.5%. Performance testing revealed that the polyester had a molecular weight of approximately 20 kg/mol, a glass transition temperature of 129.7 °C, an onset decomposition temperature of 290 °C, and a tensile strength of 42.8 MPa. These results demonstrate high thermal stability and mechanical strength, indicating the potential for expanding the applications of aliphatic polycarbonate materials.

## 1. Introduction

In recent years, with the excessive use of fossil resources, there has been a continuous and significant increase in CO_2_ emissions, leading to severe environmental issues [1,2,3]. Under this background, the reduction and capture utilization of CO_2_ have garnered widespread attention in the industry [4,5,6,7]. In fact, CO_2_ is not only a typical greenhouse gas but also a renewable C1 resource with abundant sources and a low cost, making it highly valuable for various applications. Currently, several valuable methods and technologies for CO_2_ utilization have been explored, including application as a supercritical solvent and refrigerant [8,9], food preservatives [10], and raw materials for preparation of urea [11], methanol [12], isocyanate [13], methane [14], CO-rich syngas [15], and polycarbonates, etc. [16]. Among these utilization pathways, the preparation of aliphatic polycarbonates using CO_2_ and epoxides as monomers provided a feasible approach with good application prospects for the fixation and high-value utilization of carbon dioxide [17,18]. Aliphatic polycarbonates (APC), compared to traditional aromatic polycarbonates (PC), usually have better biodegradability and biocompatibility and can be widely used as surgical sutures, bone fixation materials, and drug release carriers in biomedical emerging fields [19,20,21].

Under catalytic action, polycarbonates can be directly prepared through the alternating copolymerization of carbon dioxide and epoxides. Compared to traditional methods such as direct phosgene condensation [22], ester exchange [23], and ring-opening of cyclic carbonates [24], this approach has advantages of abundant and inexpensive raw materials, simple synthetic process, and high operational safety, making it more promising for practical application. Currently, commonly used catalysts are organic compounds of transition metals (Zn, Co, Mn, etc.), including metal alkyls [25], metal carboxylates [26], metal porphyrins [27], metal diimine complexes [28], and SalenM(III)X (M = Cr, Al, Co), etc. [29]. Among these various catalysts, SalenM(III)X has attracted more significant attention in recent years. Compared to Al and Cr ions, Co ion has a more favorable electronic structure and acidity [30], making it better suited for the copolymerization of carbon dioxide and epoxides. Among them, SalenCo(III)X catalysts have been applied in the preparation of isotactic poly(propylene carbonate) (PPC) due to their excellent catalytic performance and simple preparation process. However, being a flexible molecular structure, PPC has some limitations in practical applications, including weak mechanical strength, low glass transition temperature (*T*_g_ < 40–50 °C), and poor weather resistance [31]. Relevant studies have shown that introducing rigid cyclic groups into the main chain of polycarbonate can restrict the free movement of segments, significantly increasing the rigidity of the molecular chains and the intermolecular interaction forces, which can effectively improve the mechanical strength and thermal resistance, etc. [32,33].

**Figure 1:**
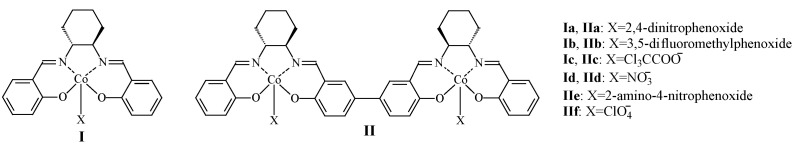
Structure of mononuclear and dinuclear cobalt complexes.

From the perspective of structure-property relationships in aliphatic polycarbonates, cyclohexene oxide, with a rigid hexagonal structure, has outstanding advantages among similar epoxides. Therefore, research on the alternating copolymerization of carbon dioxide and cyclohexene oxide has been conducted to develop a new, excellent polycarbonate material named poly(cyclohexenylene carbonate) (PCHC), which is of great significance for promoting the application of aliphatic polycarbonates [34,35]. However, cyclohexene oxide exhibits relatively low reactivity compared with other epoxides, and conventional mononuclear cobalt catalysts have difficulty achieving the desired results [36,37,38]. In this study, based on the bimetallic synergistic catalytic mechanism, a novel dinuclear cobalt catalysts was prepared and used for the alternating copolymerization of carbon dioxide and cyclohexene oxide (Figure 1). A novel PCHC material with high stereoregularity was successfully synthesized. In addition, the thermodynamic and mechanical properties were also tested, and the influence of stereoregularity on these properties is preliminarily discussed based on these results.

## 2. Results and Discussion

### 2.1. Catalytic Activity of Catalysts

Catalysts have a significant impact on the efficiency of polymerization reactions, the molecular weight and distribution of polyesters, as well as the selectivity of their stereo structures. The catalytic results of different catalysts are shown in Table 1.

It can be seen from Table 1 that the catalytic activity of binuclear cobalt complexes was significantly higher than that of mononuclear cobalt complexes. A possible reason is that the rigidly connected biphenyl groups maintain an appropriate distance between the two cobalt atoms, achieving good synergistic catalytic effects [39]. Excellent catalytic activity can be realized without the use of bis(triphenylphosphine)iminium chloride (PPNCl) as a co-catalyst, which makes the catalytic system simpler. On the other hand, mononuclear cobalt complex catalysts exhibited almost no catalytic activity in the polymerization reaction without the co-catalyst PPNCl.

Axial coordinating ions have a significant impact on the electronic properties of the central cobalt ion, thereby affecting the catalytic activity [40]. When the coordinating ions were 2,4-dinitrophenoxide, 3,5-bis(trifluoromethyl)phenoxide, and trichloroacetic acid ion, etc., both mononuclear and dinuclear cobalt complexes exhibited relatively high catalytic activity and stereochemical selectivity. The reason may be that these ions all have strong electron-withdrawing abilities, which can keep the central cobalt atom in an electron-deficient state and maintain a stable Co(III) structure, resulting in a high and persistent catalytic activity. Conversely, when the weak nucleophiles ClO_4_^−^ acted as an axial coordinating ion, it basically lacked the ability to catalyze the copolymerization reaction and could not even generate polymers. Although NO_3_^−^ has some nucleophilic ability, its ease of losing electrons may cause part of Co(III) ions to be reduced to Co(II) ions and became inactive, leading to relatively lower catalytic activity [41].

### 2.2. Influence of Operation Conditions

Operating conditions, especially the pressure of CO_2_ and temperature, have a significant impact on the alternating copolymerization reaction rate of carbon dioxide and cyclohexene oxide, as well as the molecular weight and distribution of the polycarbonate products. In this study, the polymerization results under different reaction pressure and temperature conditions were investigated using the most effective dinuclear cobalt complex **IIa** as a catalyst under the same material ratio and reaction time. The results are shown in Table 2.

From Table 2, it can be observed that the variations in temperature and CO_2_ pressure had a significant impact on the polymerization reaction. At room temperature (25 °C), increasing the CO_2_ pressure from 2.0 MPa to 4.0 MPa resulted in an increase in TOF from 133 h^−1^ to 166 h^−1^, indicating a significant improvement in catalyst effect. Additionally, the *ee* value of the polycarbonate increased from 72.5% to 93.5%, indicating an enhanced stereo-regularity and greater specificity of the polymerization reaction. As for temperature, although raising the reaction temperature can also significantly increase the TOF of the catalyst—for instance, at a CO_2_ pressure of 2.0 MPa, increasing the temperature from 25 °C to 45 °C could elevate the TOF to 2.16 times the original value—the stereo-regularity of the polyester inevitably decreased in some extent. This is because the energy difference between the two enantiomeric structures is small and at higher temperatures, the rate of the unfavorable reaction increases significantly, leading to increased randomness in the polycarbonate and a decrease in *ee* value. Overall, high pressure and low temperature conditions are more favorable for obtaining highly stereo-regular PCHC.

### 2.3. Analysis of the Structure and Properties of PCHC

#### 2.3.1. Stereo-Regularity

**Figure 2:**
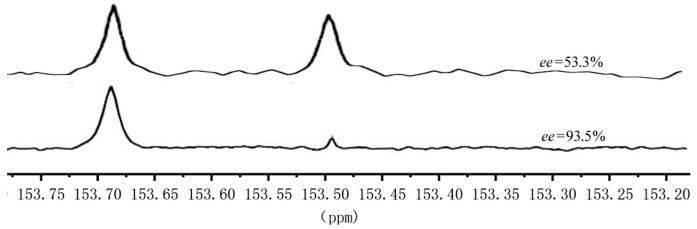
Local magnification of the ^13^C NMR spectrum of different stereo-regularity PCHC.

Besides determining the *ee* value of the hydrolysis products of PCHC, ^13^C NMR is also a direct and effective means for assessing the stereo-regularity of PCHC [42]. The carbonyl carbons of PCHC in different chemical environments exhibit distinctive chemical shifts in the ^13^C NMR spectrum. Peaks at 153.45–153.50 ppm represents the syndiotactic structure, while the peak at 153.65–153.70 ppm belongs to the isotactic structure. Furthermore, a relatively larger peak area at 153.65–153.70 ppm indicates higher stereo-regularity of the PCHC material. Figure 2 displays the ^13^C NMR spectra of two representative samples, sample A (Table 1, Entry 4) and sample B (Table 2, Entry 3).

From Figure 2, it can be observed that the signal strengths in 152.8–153.5 ppm and 153.65–153.70 ppm of sample A are not significantly different. As for sample B, the signal of the meso structure carbonyl peak in the range of 153.45–153.50 ppm was relatively weak, while the peak at 153.65–153.70 ppm corresponding to the isotactic structure carbonyl carbon was prominent, indicating the dominance of the isotactic structure in the molecule. After hydrolyzing the two PCHC samples and measuring the excess enantiomer content of the mixed alcohols using a chiral gas chromatography, the *ee* value was 53.3% and 93.5% respectively, demonstrating that catalyst **IIa** has a more excellent asymmetric catalytic effect.

#### 2.3.2. Thermal Properties

**Figure 3:**
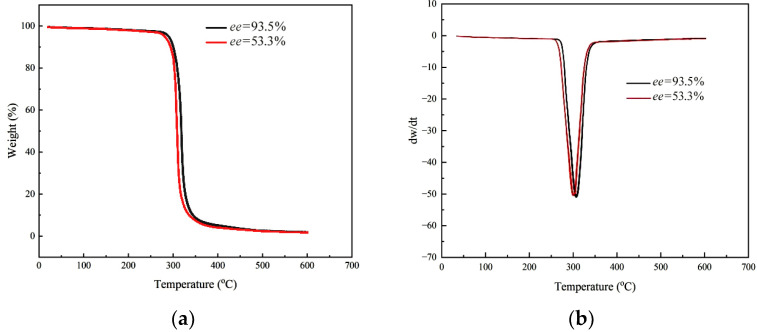
TGA (**a**) and DTG (**b**) curves of two PCHC samples with different *ee* value.

To investigate the influence of stereo-regularity on PCHC material, sample A and sample B were detected, respectively. The thermal gravimetric analysis (TGA) results are shown in Figure 3.

From Figure 3, it can be observed that the two PCHC samples with different *ee* values exhibited different initial decomposition temperatures (*T*-5w%), which were 280 °C and 290 °C, respectively. These values were significantly higher than that of PPC with a similar molecular weight (217 °C). Additionally, both of them showed a narrow temperature range of thermal decomposition, approximately 10 °C, indicating high purity and low content of ether segments in the two samples. The DTG curves revealed that the maximum decomposition temperatures (*T*-50w%) of the two PCHC samples were 300 °C and 309 °C, respectively, with sharp exothermic peaks. These observations suggested that the PCHC materials possess better thermal stability than PPC. Furthermore, PCHC with higher stereo-regularity (*ee* value) exhibited superior thermodynamic properties, which can be attributed to the more ordered molecular arrangement and stronger intermolecular forces.

**Figure 4:**
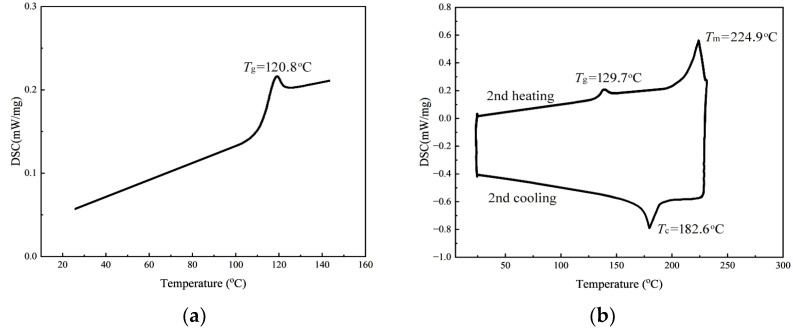
DSC curves of two PCHC samples with different *ee* values: (**a**) *ee* = 53.3%, (**b**) *ee* = 93.5%.

To validate this hypothesis, we conducted further analysis using differential scanning calorimetry (DSC) on the two samples. The results are shown in Figure 4.

According to the literature, PCHC tends to exhibit crystallization behavior when the *ee* value exceeds 91% [43]. The study of DSC can provide valuable insights into the crystallization behavior and thermal properties. For PCHC samples with an *ee* value of 53.3%, the stereo-regularity was relatively low, resulting in the absence of crystallization. Therefore, in Figure 4a, only the glass transition temperature (*T_g_*) can be observed. From Figure 4b, it can be seen that the PCHC sample with an *ee* value of 93.5% indeed exhibits crystallization behavior observed from second cooling curve. No crystallization peak appears in the second heating curve; the reason is because crystallization has been induced by the first heating program, so there was no crystallization peak appeared in the second heating process, only a melting endotherm peak. However, since the stereo-regularity did not reach 96–100%, a glass transition phenomenon still occurred with a value of 129.7 °C, which is consistent with the findings reported in reference [43]. The enthalpy change of the PCHC can be obtained by integrating the corresponding DSC curve with heating time [44]. The results revealed a distinct endothermic peak at a melting point of 224.9 °C, with a melting enthalpy (∆*H*_m_) of 20.691 J/g. During the cooling process, an exothermic peak appeared around 182.6 °C, indicating the occurrence of crystallization, with a crystallization enthalpy (∆*H*_c_) of −17.312 J/g.

#### 2.3.3. Mechanical Properties

**Figure 5:**
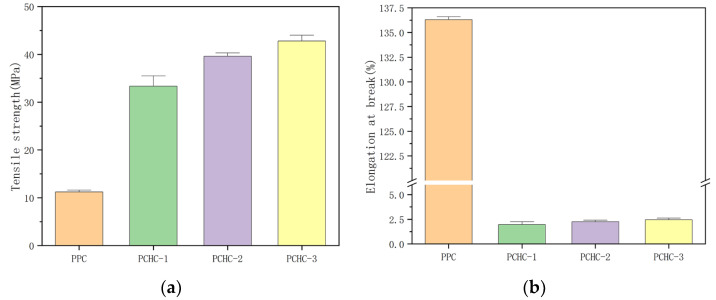
Tensile strength (**a**) and elongation at break (**b**) of PCHC samples with different *ee* values.

Mechanical properties are important characteristics of structural materials and have a significant impact on their application fields and service life [45,46]. We conducted tensile strength and elongation at break measurements on the PCHC samples with different *ee* values (the samples with *ee* value of 53.3%, 72.5% and 93.5% were named PCHC-1, PCHC-2 and PCHC-3) and compared these values with those of the currently used PPC material. The results are shown in Figure 5.

From Figure 5, it can be observed that the comparative sample of PPC is a ductile material with a high elongation at break of 136.3%. However, its tensile strength is low, only 11.2 MPa, indicating a deficiency in material strength. In contrast, the PCHC material prepared in this study, with the introduction of bulky hexagonal ring structures in the main chain of polymer molecule, exhibited significantly improved strength and rigidity compared to PPC. Even the low stereo-regularity PCHC with an *ee* value of only 53.3% showed a high tensile strength of 33.0 MPa, nearly three times that of PPC material. Meanwhile, the tensile strength increased significantly with the increase of the *ee* value, eventually reaching 42.8 MPa. However, the elongation at break did not show significant improvement, which may be attributed to the rigid nature of PCHC material. So, it can be drawn that increasing the stereo-regularity of PCHC is beneficial for enhancing its mechanical properties. However, it should be noted that PCHC is a rigid material with poor deformability, exhibiting an elongation at break of only about 2.5%, and a relatively brittle texture. If the properties of PCHC and PPC can be combined, it is possible to obtain a composite polycarbonate material with both satisfactory strength and toughness.

## 3. Materials and Methods

### 3.1. Chemicals

Carbon dioxide, oxygen, and nitrogen were purchased from Henan Yuanzheng Special Gas Co., Ltd. (Zhengzhou, China). Cyclohexene oxide was provided by Henan Shenma Nylon Co., Ltd. (Pingdingshan, China). Anhydrous cobalt acetate, 3,5-bis(trifluoromethyl)nitrophenol, 2-amino-4-nitrophenol, trichloroacetic acid, and 2,4-dinitrophenol bis(triphenylphosphine)ammonium chloride were all analytical grade and purchased from Shanghai McKlin Technology Co., Ltd. (Shanghai, China). Poly(propylene carbonate) (*M*_n_ = 100 kg/mol, *T_g_* = 49.8 °C) was obtained from Shenzhen Hongli Plastic Raw Material Co., Ltd. (Shenzhen, China). Other reagents and solvents were all analytical grade and were purchased from Tianjin ChemiO Chemical Reagent Co., Ltd. (Tianjin, China). All raw materials needed to be dried before use to reduce the moisture content. The 3,3′-diformyl-4,4′-dihydroxy-1,1′-biphenyl and salicylaldehyde condensation product were prepared according to references [47,48], respectively, and their structures were confirmed by FTIR and NMR characterization (Figures S1–S6). The monometallic cobalt catalysts **Ia**–**Id** used for comparison were prepared according to references [49,50].

### 3.2. Synthetic Route

#### 3.2.1. Catalyst of Dinuclear Cobalt Complexes

**Figure 6:**
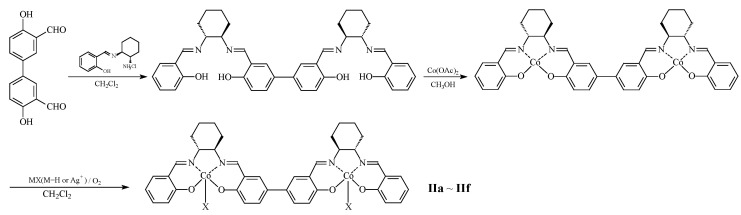
Synthetic route of dinuclear cobalt complex.

The preparation of the bimetallic catalyst started with materials (S,S)-cyclohexane-1,2-diamine aldehyde condensation product and 3,3′-diformyl-4,4′-dihydroxy-1,1′-biphenyl as starting reagents, and it underwent condensation, salt formation, oxidation, and ion exchange to obtain the desired catalyst. The preparation route is shown in Figure 6.

#### 3.2.2. Poly(cyclohexenylene carbonate)

**Figure 7:**
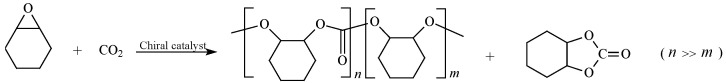
Synthetic route of stereoregular PCHC.

The preparation of isotactic poly(cyclohexene carbonate) involved carbon dioxide and cyclohexene oxide as starting materials. Under the action of a chiral catalyst, the mixture underwent alternating copolymerization under high-pressure conditions to obtain the desired PCHC. The preparation route is shown in Figure 7.

### 3.3. Synthesis of Compounds

#### 3.3.1. Salen Ligand

A 100 mL round-bottom flask equipped with a magnetic stir bar was placed in a low-temperature cooling bath, and stirring was initiated. The temperature was controlled below 0 °C. 3,3′-diformyl-4,4′-dihydroxy-1,1′-biphenyl (0.24 g, 1.00 mmol), condensate of (*S*,*S*)-cyclohexane-1,2-diamine hydrochloride, and salicylaldehyde (0.485 g, 2.00 mmol) were dissolved in 60 mL of CH_2_Cl_2_. Then, triethylamine (0.55 mL, 4.00 mmol) and a small amount of 5A molecular sieve were added. The mixture was allowed to react at room temperature for 24 h and then filtered under vacuum. The filter cake was washed with an appropriate amount of CH_2_Cl_2_ before collecting the filtrate. The crude product was obtained by vacuum distillation of the filtrate and purified by column chromatography (dry loading; silica gel column; eluent: petroleum ether/ethyl acetate = 10/1), resulting in a golden yellow powder compound with a yield of 85.6%. IR (KBr, cm^−1^) ν: 3455, 3241, 2932, 2740, 1649, 1498, 1456, 1276, 1145, 1035, 823, 759, 660; ^1^H NMR (CDCl_3_, 400 MHz) δ 13.61 (s, 2H), 13.13 (s, 2H), 8.38 (s, 2H), 8.23 (s, 2H), 7.22 (s, 2H), 7.13 (s, 2H), 6.89 (m, 2H), 6.85 (s, 2H), 6.76 (s, 2H), 3.62–3.55 (m, 2H), 3.32–3.26 (m, 2H), 2.02–1.81 (m, 4H), 1.80–1.51 (m, 4H), 1.50–1.31 (m, 12H). ^13^C NMR (DMSO-*d*_6_, 100 MHz) δ 162.8, 162.4, 160.8, 160.7, 132.7, 132.3, 130.5, 129.7, 120.2, 119.8, 119.6, 119.4, 118.8, 117.0, 67.6, 67.2, 32.9, 32.7, 24.2, 24.1. The spectra of IR, ^1^H-NMR and ^13^C-NMR were listed in Figures S7–S9.

#### 3.3.2. SalenCo(II) Complex

Under N_2_ protection, salen ligand (0.321 g, 0.50 mmol) and 5 mL of CH_2_Cl_2_ were added to a 150 mL three-neck flask. Then, the flask was placed in a low-temperature cooling bath with magnetic stirring. The solution of 0.18 g anhydrous cobalt acetate in 30 mL of CH_3_OH was slowly added dropwise to the flask within 20 min and stirred for 30 min. Then, the precipitate was filtered out and washed with a small amount of CH_3_OH. The filter cake was vacuum dried at 60 °C for 24 h, resulting in the formation of a brick-red solid product with a yield of 93.1%. ^1^H NMR (CDCl_3_, 400 MHz) *δ* 8.38 (s, 2H), 8.23 (s, 2H), 7.22 (s, 2H), 7.13 (s, 2H), 6.89 (m, 2H), 6.85 (s, 2H), 6.76 (s, 2H), 3.62–3.54 (m, 2H), 3.32–3.25 (m, 2H), 2.02–1.80 (m, 4H), 1.80–1.51 (m, 8H), 1.50–1.32 (m, 4H). ^13^C NMR (DMSO-*d*_6_, 100 MHz) *δ* 164.6, 164.1, 136.4, 135.7, 132.5, 129.5, 128.7, 128.3, 128.1, 127.2, 125.1, 124.0, 117.1, 116.0, 51.7, 51.4, 30.1, 30.0, 23.8. The spectra of ^1^H-NMR and ^13^C-NMR were listed in Figures S10 and S11.

#### 3.3.3. SalenCo(III) Complexes

The preparation processes of SalenCo(III) complexes were similar with minor variations. Taking the preparation of catalyst **IIa** as an example here: In a 50 mL three-neck flask, SalenCo(II) complex (0.151 g, 0.2 mmol) and 2,4-dinitrophenol (0.074 g, 0.4 mmol) were dissolved in 30 mL of purified CH_2_Cl_2_. Dry oxygen was slowly introduced, and the mixture was oxidized for 1–2 h. The solvent was then removed by rotary evaporation to obtain the crude product. Then, the crude product was taken out and dissolved in a small amount of ethyl ether, and a small amount of n-hexane was added to adjust polarity. The mixture was placed at a low temperature and kept in the dark overnight. After filtration, the precipitate was collected and vacuum dried at 60 °C for 12 h, resulting in a dark green powder with a yield of 88.2%. ^1^H NMR (CDCl_3_, 400 MHz): *δ* 8.76 (s, 2H), 8.29 (s, 2H), 7.83 (s, 2H), 7.51 (m, 2H), 7.38–7.21 (m, 8H), 6.98–6.88 (m, 4H), 6.55 (d, 2H), 2.96–2.76 (m, 4H), 2.01–1.83 (m, 4H), 1.75–1.56 (m, 8H), 1.53–1.37(m, 6H). ^13^C NMR (DMSO-*d*_6_, 100 MHz) δ 162.6, 162.2, 159.3, 141.1, 140.8, 138.9, 138.7, 136.7, 136.3, 133.2, 133.1, 131.7, 131.5, 124.7, 124.5, 122.6, 122.4, 118.4, 118.2, 117.3, 117.1, 53.2, 52.8, 52.7, 27.1, 26.9, 26.8, 23.3, 23.1. The spectra of ^1^H-NMR and ^13^C-NMR were listed in Figures S12 and S13.

#### 3.3.4. Poly(cyclohexenylene carbonate)

The high-pressure reactor was placed in a drying oven and dried at 120 °C for 2 h. Then, it was quickly removed while still hot, and it was sealed and evacuated under vacuum. Nitrogen gas was introduced to replace the atmosphere. In a nitrogen-filled glove box, a predetermined amount of catalyst and a certain proportion of cyclohexene carbonate were weighed into a 250 mL iodine flask successively. After the catalyst dissolved, the mixture was transferred to the high-pressure reactor using a syringe. CO_2_ was introduced into the reactor until the set pressure was reached (2.0–4.0 MPa). The mixture was stirred violently at a specific temperature for a certain time (controlling the conversion rate at 50–60%). Then, the mixture was cooled to room temperature, and CO_2_ was slowly released. The mixture was transferred to a 200 mL single-neck flask, and the excessive cyclohexene was removed by vacuum distillation. The precipitated solid in the flask was dissolved in a small amount of chloroform, and a few drops of dilute hydrochloric acid (2 mol/L) were added. The solution was then added to methanol and vigorously stirred to precipitate the polymer. This process was repeated several times to remove the residue catalyst, resulting in a white powder of PCHC after vacuum drying. The spectra of ^1^H-NMR was listed in Figure S14.

### 3.4. Structure and Properties Analysis of PCHC

#### 3.4.1. GPC Analysis

Molecular weight and its distribution of PCHC samples were analyzed using a PL-GPC50 gel permeation chromatography instrument (Agilen, Santa Clara, CA, USA). THF was used as the mobile phase at a flow rate of 1.00 mL/min, and the sample concentration was 5 mg/mL (standard sample: polystyrene).

#### 3.4.2. TG Analysis

Thermogravimetric analysis was carried using a TGA2 thermal analyzer (Mettler-Toledo, Greifensee, Switzerland). A total of 5.0 mg of PCHC sample was put into an alumina crucible, which was heated from 25 °C to 600 °C with a heating rate of 10 °C/min and a nitrogen flow rate of 20 mL/min.

#### 3.4.3. DSC Analysis

DSC analysis was performed using a Netzsch STA-449-F1 differential scanning calorimeter (Netzsch, Germany). The measurement was conducted under N_2_ atmosphere. The sample was heated from 25 °C to 235 °C at a rate of 10 °C/min and held at 235 °C for 10 min to eliminate the sample’s thermal history. Subsequently, the temperature was decreased to 25 °C at a rate of 10 °C/min. Afterward, the second heating program was performed, where the temperature was still increased from 25 °C to 235 °C at a rate of 10 °C/min but without any holding time, and decreased from 235 °C to 25 °C at a rate of 10 °C/min. The heat flow data were recorded and used to plot the heat flow curve.

#### 3.4.4. Determination of *ee* Value

**Figure 8:**
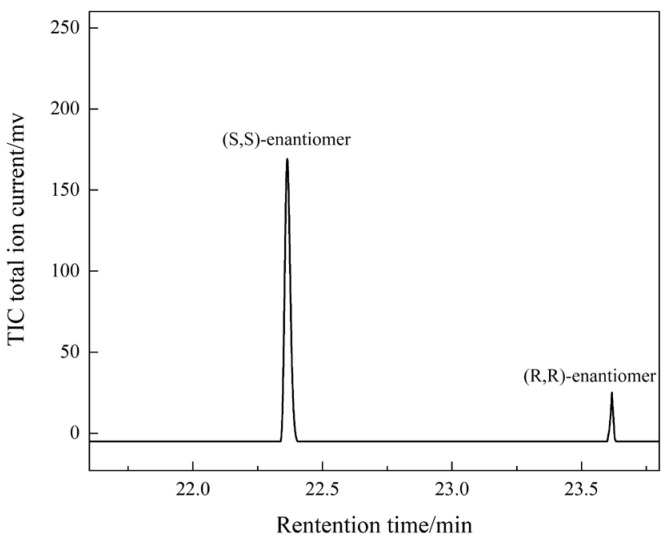
The chiral chromatogram of PCHC hydrolysis products.

A total of 120 mg of PCHC sample and 15 mL THF were added to a 100 mL round-bottom flask. Then, 2 mL of methanol and 4 mL of 3 mol/L NaOH solution were added after the PCHC dissolved. The mixture was stirred at room temperature for 8 h and the pH of the solution was subsequently adjusted to neutral by adding a small amount of 2 mol/L HCl. The aqueous phase was extracted with ethyl acetate (5 mL × 3). Then, the organic phase was dried with anhydrous MgSO_4_ overnight. The solvent was removed by rotary evaporation, and the residue was separated by column chromatography (silica gel column, petroleum ether/ethyl acetate = 10/1). An amount of 79 mg of a mixture of chiral diols was obtained after removing the solvent. The *ee* value of the chiral diols mixture was analyzed using an Agilent HP 19091G-B213 chiral gas chromatograph. The operation conditions were as follows: injection temperature of 250 °C, hydrogen flame detector temperature of 250 °C, vaporizer temperature of 260 °C, and programmed heating from 100 °C to 120 °C with a heating rate of 10 °C/min. The *ee* values were calculated by the Equation (1).

(1) $$ee=\frac{\left[S,S]-[R,R\right]}{\left[S,S]+[R,R\right]}\times 100\%$$

where [S,S] and [R,R] are the mass content of (*S*,*S*)-enantiomer and (*R*,*R*)-enantiomer of 1,2-cyclohexanediol, respectively. Since the absolute correction factors for the two components are identical, the mass percentage content can be replaced by the percentage of peak area. A typical chiral gas chromatogram is shown in Figure 8.

#### 3.4.5. Mechanical Performance Test

The mechanical properties of polyester were tested on a CMT6103 universal material testing machine (ZWICK, Ulm, Germany) according to the reference [51]. At room temperature, the PCHC sample was fully dissolved in a small amount of CH_2_Cl_2_. The viscous liquid was then evenly spread in a rectangular mold and left to stand for the solvent to evaporate under vacuum drying at 25 °C, resulting in a thin film whose thickness was about 0.5 mm. The film was cut into uniform rectangular shapes of 1.0 cm × 6.0 cm. The clamp distance was 50 mm, the test was conducted at a speed of 100 mm/min, and the measurements were taken three times using three samples of the same specifications to obtain an average value.

## 4. Conclusions

Starting from the bimetallic synergistic catalytic mechanism for alternating copolymerization of carbon dioxide and epoxides, a series of chiral bimetallic cobalt complexes catalysts with different axial coordinating ions was designed and prepared for the alternating copolymerization of carbon dioxide and cyclohexene oxide to produce stereoregular PCHC in this paper. The results revealed that catalysts with strong electron-withdrawing coordinating ions exhibited better catalytic activity, enabling efficient alternating copolymerization at room temperature and ensuring a higher stereo-selectivity during the copolymerization process. It is also observed that high temperature and low pressure contribute to the improvement of isotacticity. Subsequent property tests indicated that PCHC with higher isotacticity exhibits improved thermodynamic and mechanical properties, with a glass transition temperature of over 120 °C, which significantly expanded the temperature range for the application of polycarbonates. Furthermore, the higher mechanical strength of PCHC might provide an effective approach for improving the mechanical properties of PPC. However, further in-depth research is needed to explore these aspects.

## Figures and Tables

**Table 1:** Result of copolymerization catalyzed by different catalysts (*P* = 2.0 MPa, *T* = 25 °C).

Entry	Catalyst	CHO/Cat/PPNCl (Molar Ratio)	Time (h)	TOF (h^−1^)	*M*_n_ (kg/mol)	PDI (*M*_w_/*M*_n_)	*ee* (%)
1	**IIa**	1000/1/1	3	137	19.2	1.24	74.2
2	**IIa**	1000/1/0	3	133	18.3	1.19	72.5
3	**IIb**	1000/1/0	3	126	8.4	1.20	67.3
4	**IIc**	1000/1/0	3	84	19.8	1.22	53.3
5	**IId**	1000/1/0	3	58	10.9	1.31	37.3
6	**IIe**	1000/1/0	3	92	11.7	1.23	63.9
7	**IIf**	1000/1/0	12	3	—	—	—
8	**Ia**	500/1/0	6	—	—	—	—
9	**Ia**	500/1/1	6	73	13.2	1.27	51.1
10	**Ib**	500/1/1	6	69	13.9	1.27	53.7
11	**Ic**	500/1/1	6	58	12.1	1.28	48.7
12	**Id**	500/1/1	6	34	6.6	1.41	41.1

**Table 2:** Results of copolymerization catalyzed by **IIa** under different pressures and temperatures.

Entry	Temperature (°C)	Pressure (MPa)	TOF (h^−1^)	*M*_n_ (kg/mol)	PDI (*M*_w_/*M*_n_)	*ee* (%)
1	25	2.0	133	19.2	1.19	72.5
2	25	3.0	142	19.5	1.17	83.6
3	25	4.0	166	20.1	1.16	93.5
4	35	2.0	187	19.5	1.22	65.1
5	35	3.0	251	21.1	1.19	80.8
6	35	4.0	318	21.8	1.17	88.1
7	45	2.0	287	20.3	1.22	65.2
8	45	3.0	354	22.7	1.19	76.3
9	45	4.0	462	24.6	1.18	84.6

**Fi** n(CHO): n(CHO): n(**IIa**) = 1000, Time = 3 h.

## Data Availability

The data presented in this study are available upon request from the corresponding author.

## Supplementary Materials

The following supporting information can be downloaded at: https://www.mdpi.com/article/10.3390/molecules28135235/s1, Figure S1: FTIR spectrum of 3,3′-diformyl-4,4′-dihydroxy-1,1′-biphenyl; Figure S2: ^1^H NMR spectrum of 3,3′-diformyl-4,4′-dihydroxy-1,1′-biphenyl; Figure S3: ^13^C NMR spectrum of 3,3′-diformyl-4,4′-dihydroxy-1,1′-biphenyl; Figure S4: FTIR spectrum of salicylaldehyde condensation product; Figure S5: ^1^H NMR spectrum of salicylaldehyde condensation product; Figure S6: ^13^C NMR spectrum of salicylaldehyde condensation product; Figure S7: FTIR spectrum of the salen ligand; Figure S8: ^1^H NMR spectrum of the salen ligand; Figure S9: ^13^C NMR spectrum of the salen ligand; Figure S10: ^1^H NMR spectrum of the salenCo(II) complex; Figure S11: ^13^C NMR spectrum of the salenCo(II) complex; Figure S12: ^1^H NMR spectrum of the salenCo(III) complex (**IIa**); Figure S13: ^13^C NMR spectrum of the salenCo(III) complex (**IIa**); Figure S14: ^1^H NMR spectrum of PCHC.
