# Supplementary material for: Study of Preparation and Properties of Stereoregular Poly(cyclohexenylene carbonate)
Source: Molecules. 2023 Jul 5;28(13):5235. doi: 10.3390/molecules28135235 (PMC10343680; doi:10.3390/molecules28135235)
Supplement: Supplementary file 1 [file molecules-28-05235-s001.zip › molecules-2486202-supplementary.pdf]

Spectra of catalyst **IIa** and its intermediates

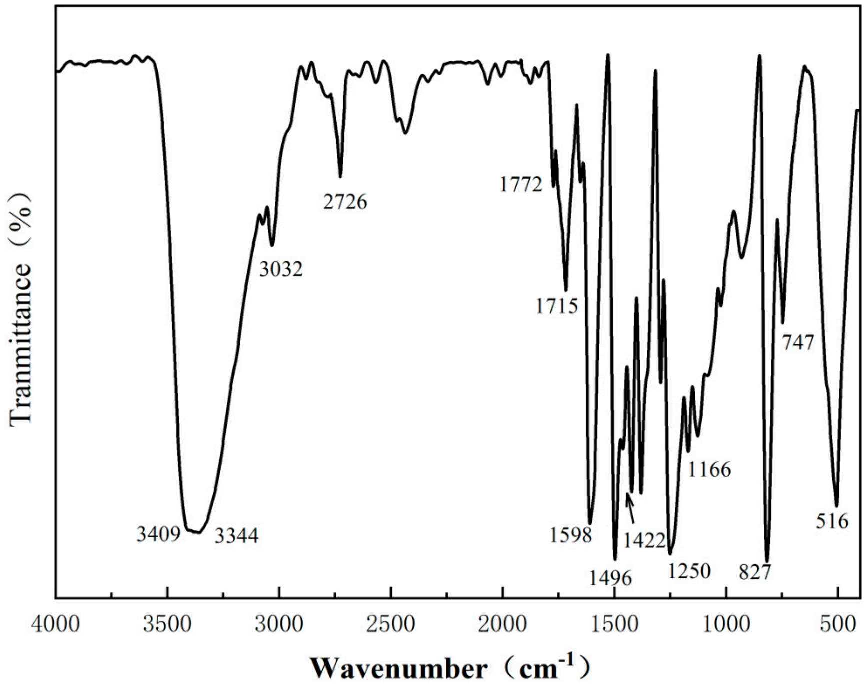

Figure S1 FTIR spectrum of 3,3’-diformyl-4,4’-dihydroxy-1,1’-biphenyl

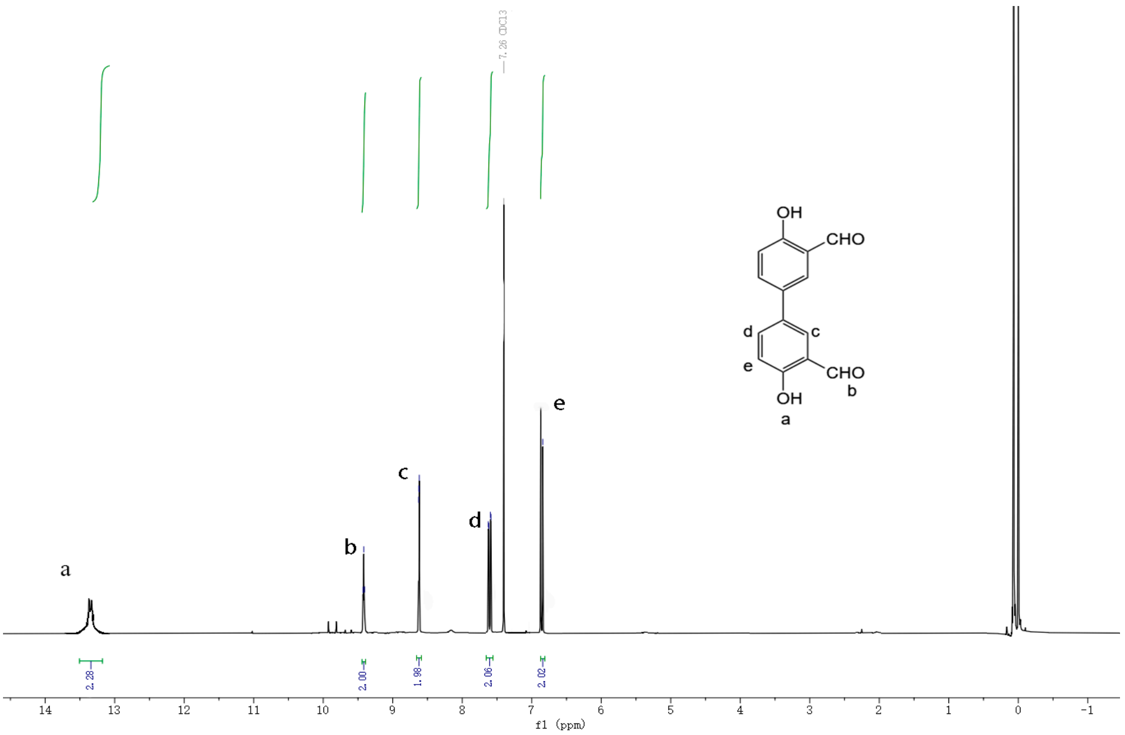

Figure S2 ^{1}H NMR spectrum of 3,3’-diformyl-4,4’-dihydroxy-1,1’-biphenyl

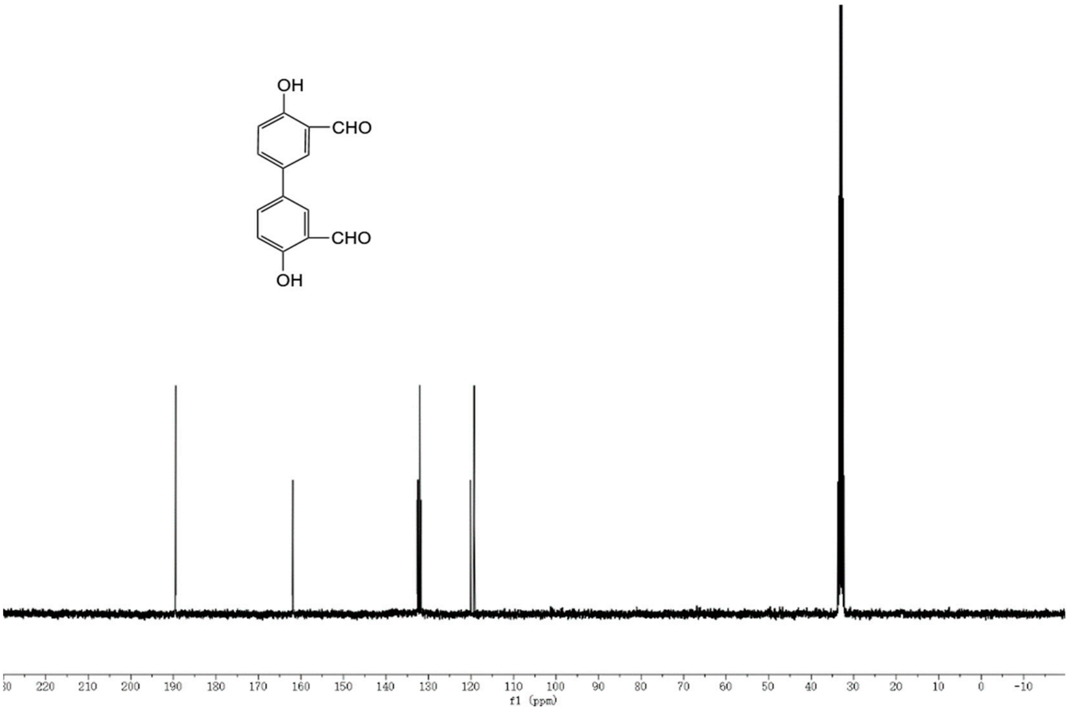

Figure S3 ^{13}C NMR spectrum of 3,3'-diformyl-4,4'-dihydroxy-1,1'-biphenyl

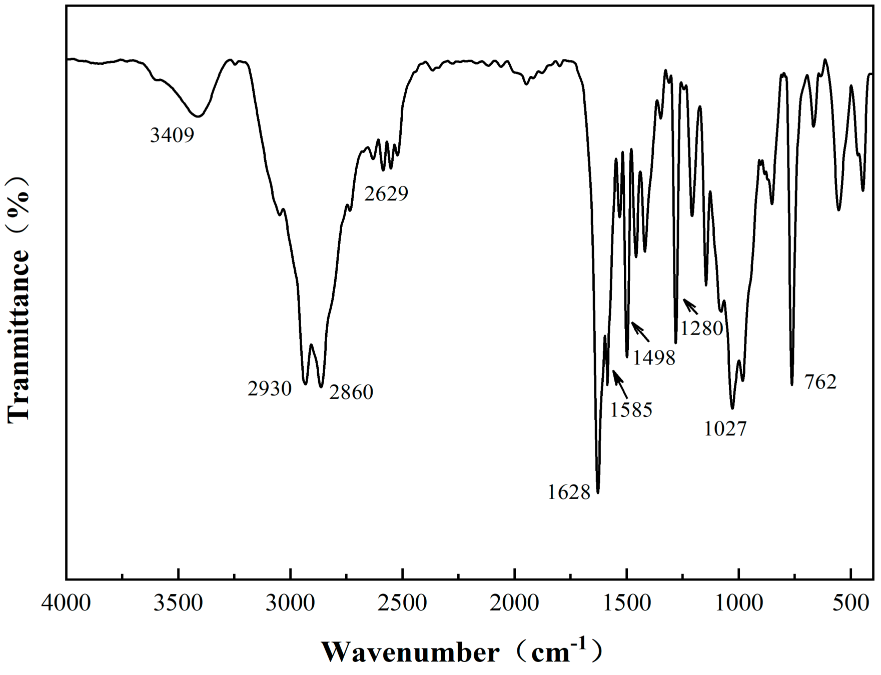

Figure S4 FTIR spectrum of salicylaldehyde condensation product

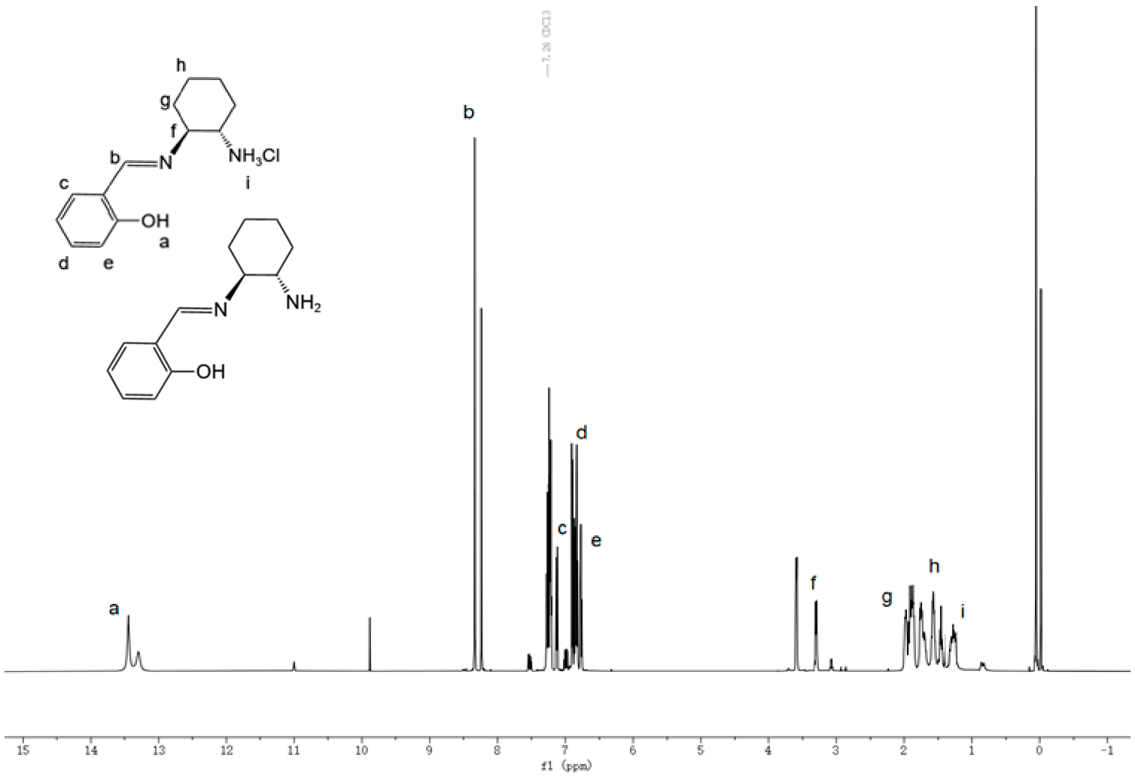

Figure S5 ^{1}H NMR spectrum of salicylaldehyde condensation product

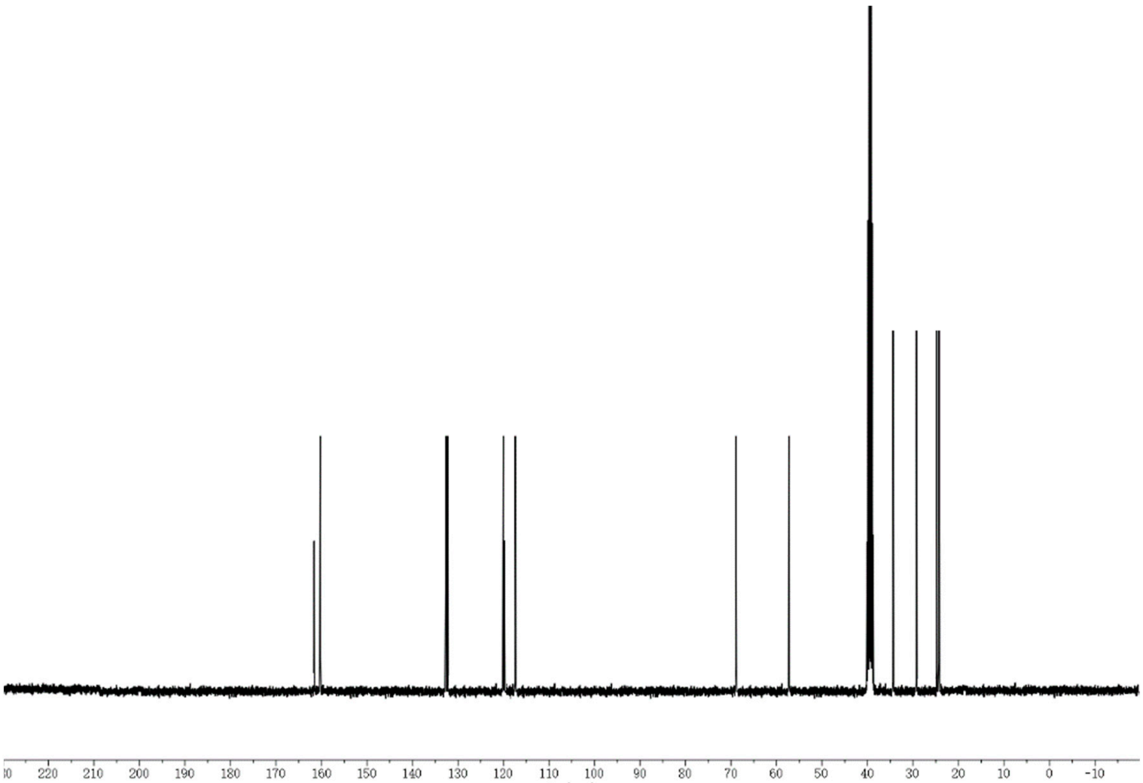

Figure S6 ^{13}C NMR spectrum of salicylaldehyde condensation product

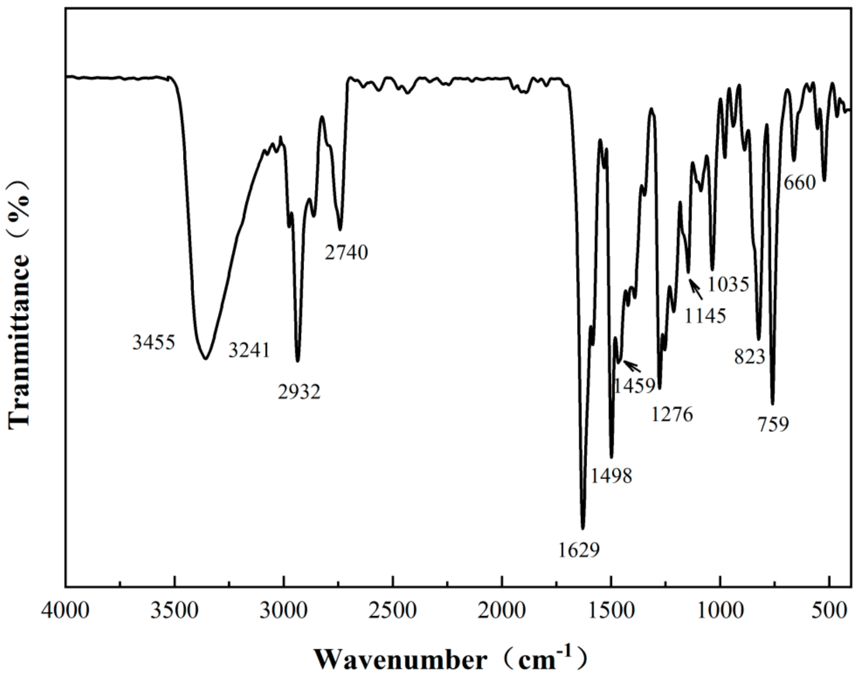

Figure S7 FTIR spectrum of the salen ligand

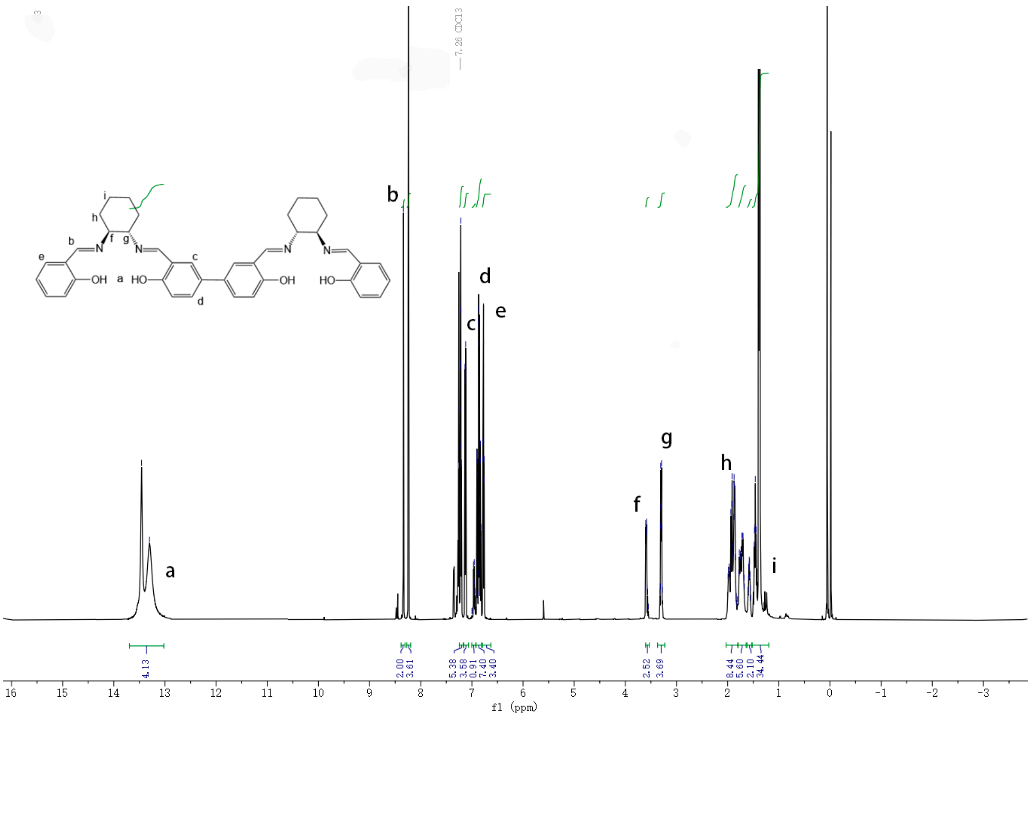

Figure S8 ^{1}H NMR spectrum of the salen ligand

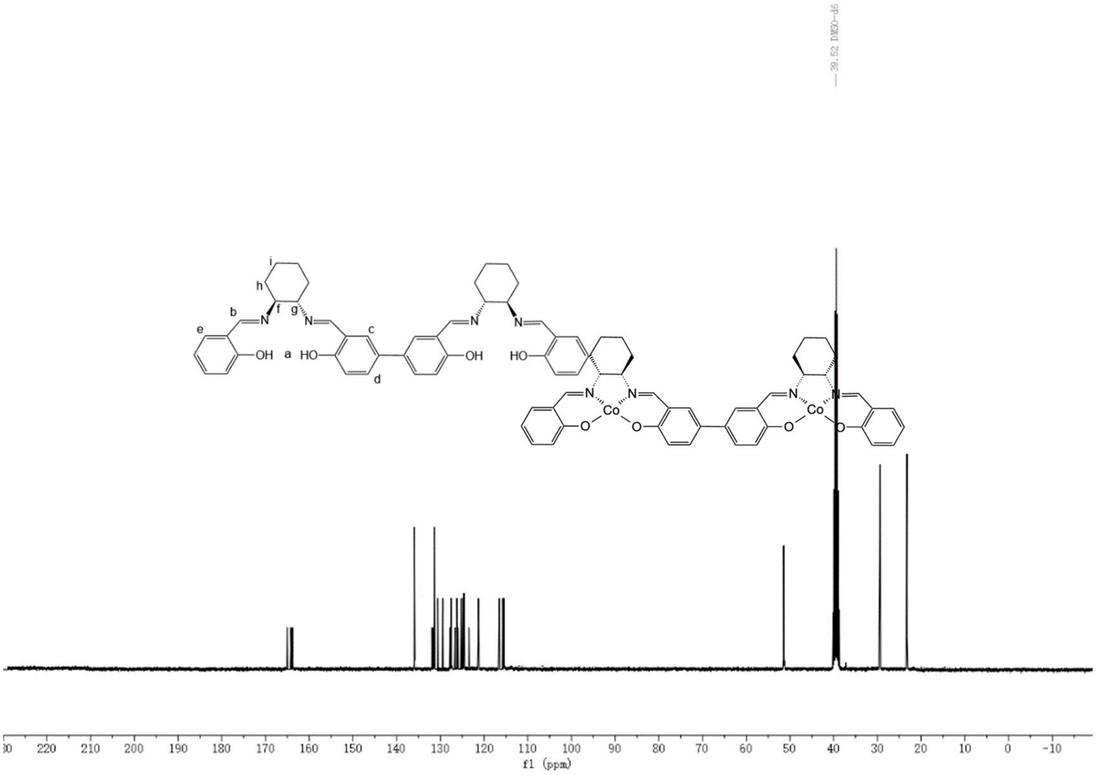

Figure S9 ^{13}C NMR spectrum of the salen ligand

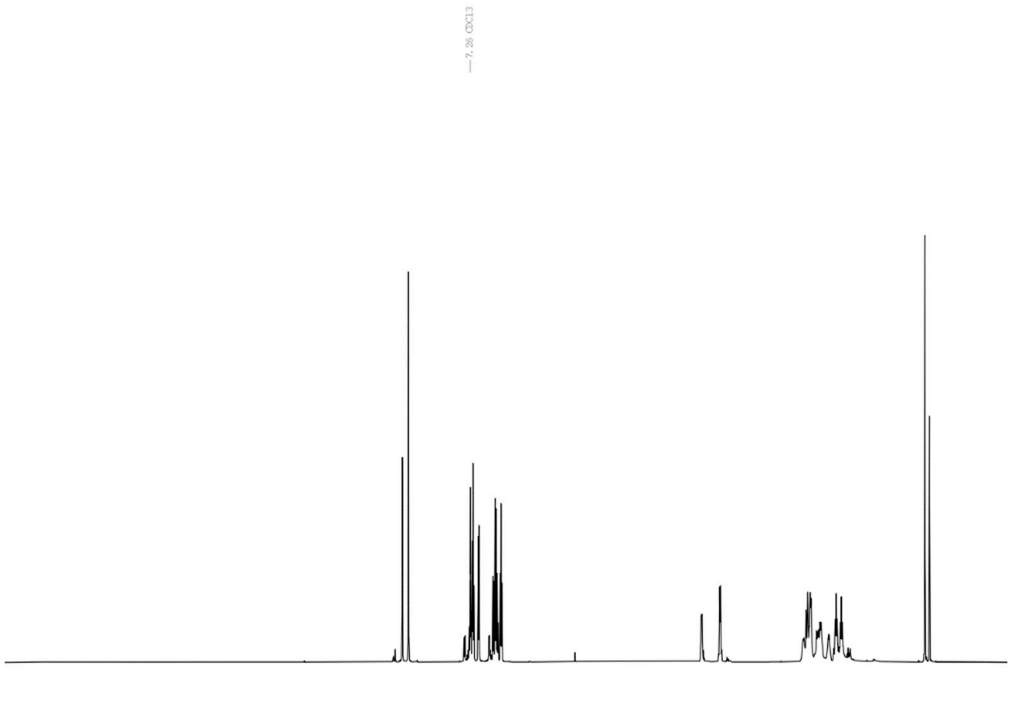

Figure S10 ^{1}H NMR spectrum of the salenCo(II) complex

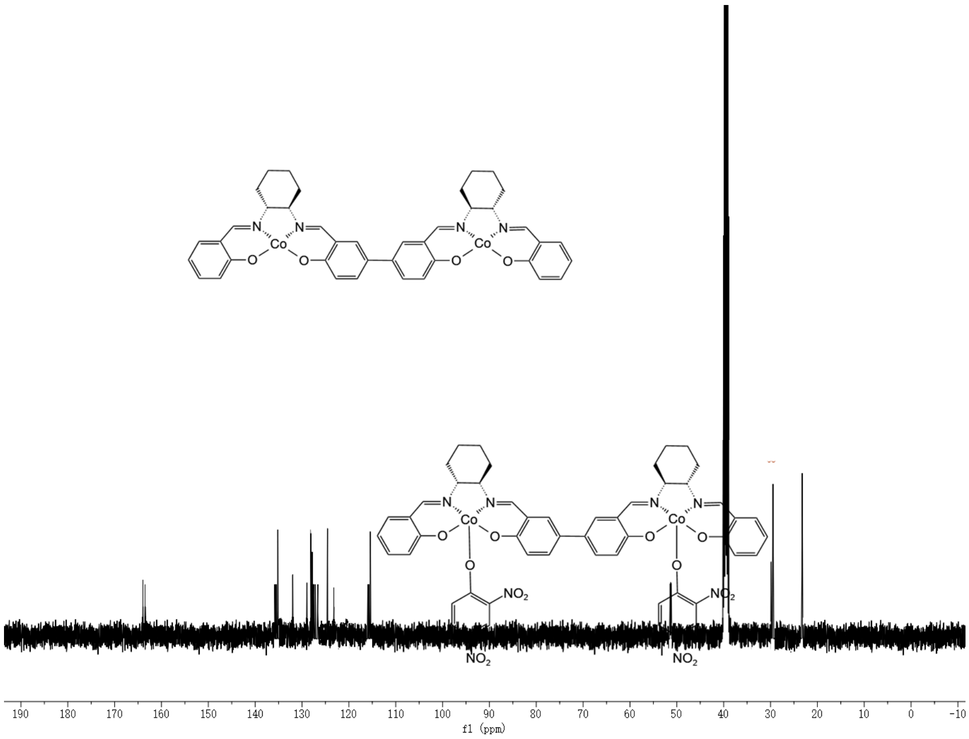

Figure S11 ^{13}C NMR spectrum of the salenCo(II) complex

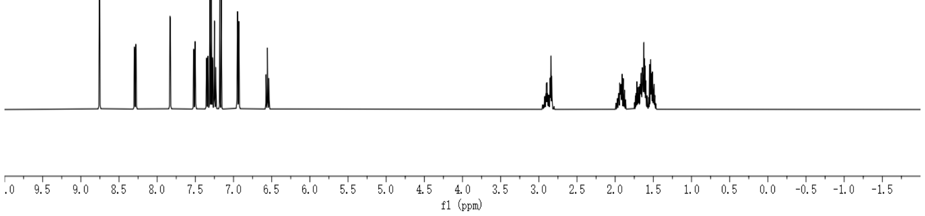

Figure S12 ^{1}H NMR spectrum of the salenCo(III) complex (**IIa**)

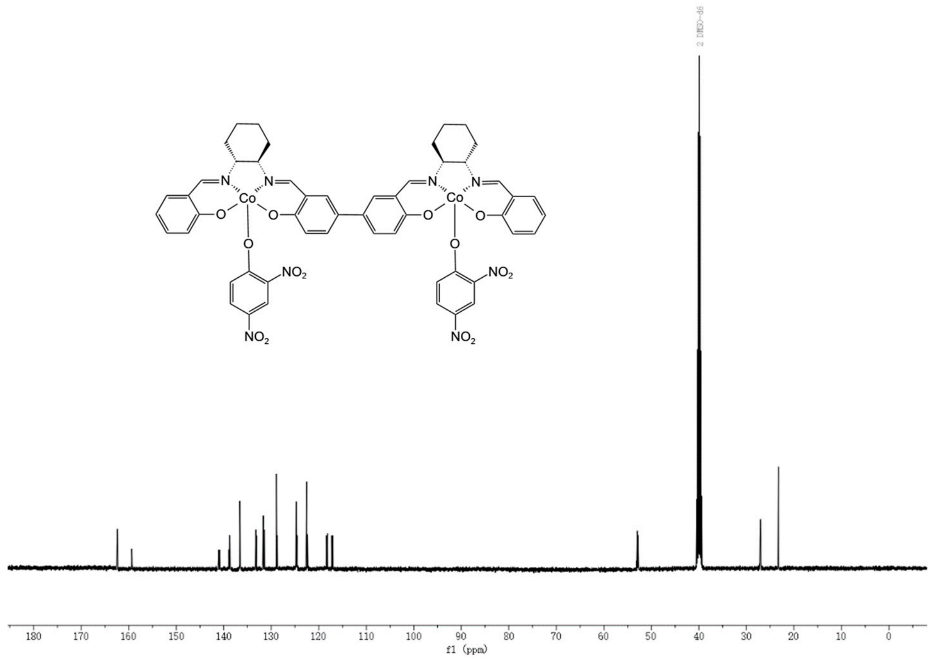

Figure S13 ^{13}C NMR spectrum of the salenCo(III) complex (**IIa**)

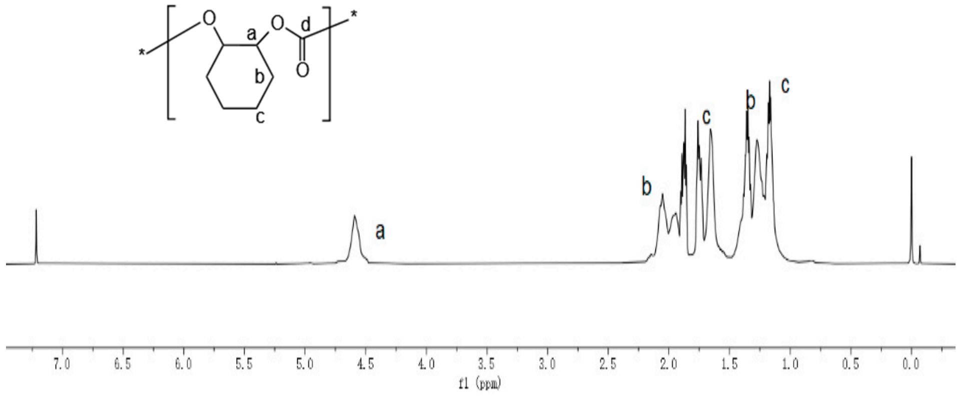

Figure S14 ^{1}H NMR spectrum of stereoregular PCHC(* stand for stereoregular)
